# Digital Twin-Based Risk Control during Prefabricated Building Hoisting Operations

**DOI:** 10.3390/s22072522

**Published:** 2022-03-25

**Authors:** Zhansheng Liu, Anxiu Li, Zhe Sun, Guoliang Shi, Xintong Meng

**Affiliations:** Department of Urban Construction, Beijing University of Technology, Beijing 100124, China; lianxiu0618@emails.bjut.edu.cn (A.L.); zhesun@bjut.edu.cn (Z.S.); shiguoliang@emails.bjut.edu.cn (G.S.); mxt0803@163.com (X.M.)

**Keywords:** digital twin, prefabricated building hoisting, safety risk control, decision visualization

## Abstract

Prefabricated buildings have advantages when it comes to environmental protection. However, the dynamics and complexity of building hoisting operations bring significant safety risks. Existing research on hoisting safety risk lacks a real-time information interaction mechanism and lacks scientific control decision-making tools based on considering the correlation between safety risks. Digital twin (DT) has the advantage of real-time interaction. This paper presents a safety risk control framework for controlling prefabricated building hoisting operations based on DT. In the case of considering the correlation of the safety risk index of hoisting, the safety risk hierarchy model of hoisting is defined in the process of building the DT model. The authors have established a Bayesian network model into the process of the integrated analysis of the digital twin mechanism model and monitoring data to realize the visualization of the decision analysis process of hoisting safety risk control. The key degree of the indirect inducement variable to direct inducement variable was calculated according to probability. The key factor leading to the occurrence of risk was found. The effectiveness of the hoisting safety risk control method is verified by a large, prefabricated building project. This method provides decision tools for hoisting safety risk control, assists in formulating effective control schemes, and improves the efficiency of information integration and sharing.

## 1. Introduction

The traditional construction industry has the disadvantages of extensive construction mode, waste of resources, frequent safety accidents, and low intelligence level [1]. Integrating modern information technology into the current practice of the construction industry has promoted the rapid development of new building industrialization represented by prefabricated buildings. Prefabricated building is a new construction method, which involves many new technologies in the construction stage [2]. Such a new construction method has the advantages of resource-saving, environmental protection, and a short construction period, which is an important way to transform the traditional construction mode to the modern industrialized construction mode [3]. Unfortunately, due to the low level of professional skills of construction workers, the construction stage of prefabricated buildings has also become a stage of frequent safety accidents. 

Prefabricated construction processes contain the transportation stage, hoisting stage, and installation stage. The hoisting stage is the most critical part of various prefabricated construction processes. Hoisting operations have the following characteristics [4]: (1) prefabricated components with large weight and high-altitude operation; (2) the hoisting operation is a combined movement, with many and scattered dangerous points; (3) large range of movement; (4) cooperation of multiple working types; (5) the influence relationship between various hoisting safety risk factors is complex. In the hoisting stage, safety accidents such as the overturning of tower cranes, rope breaking, collision, decoupling, and the falling of components occur easily [5]. The reasons for safety accidents are mostly inadequate on-site safety management, workers’ illegal operation, and inadequate equipment maintenance. Therefore, the safety control problem in the hoisting stage is worthy of in-depth study.

Safety has always been the focus of the construction industry. According to official statistics, there were 773 accidents and 904 deaths in the production of housing and municipal engineering in 2019. The object strike accidents accounted for 15.91% of the total, and the hoisting machinery injury accidents accounted for 5.43% of the total [6]. The occurrence of the two accidents is closely related to the hoisting operation. Many scholars have done a lot of research in the field of safety risk management, but there are still few research results in the field of prefabricated building safety risk management. A considerable number of scholars focus on the optimal arrangement of tower cranes [7,8], transportation scheduling [9], and motion control [10,11]. With the development of safety risk research theory and technology, the research concept of safety risk issues has also been transformed from post-analysis to pre-prevention. The research on safety issues is roughly divided into three stages [12]: the first stage focuses on the statistical analysis of safety accidents and safety measures; the second stage enters the active safety management stage, using more advanced statistical techniques to identify and evaluate the safety risk; the third stage is the real-time monitoring stage of security risks. The use of virtual reality technology for security training and the real-time collection of security risk information can prevent and control security risks in a timely fashion. However, the dynamics of construction site elements are still difficult to track, and lack effective information integration and interaction mechanisms.

At present, relying on the supervision of on-site safety management personnel to control the occurrence of safety accidents is very time-consuming. Visualization technology has been gradually applied to the safety management of construction sites. With the help of location information, imaging information, and alarm technology, workers’ behaviors and site environments (including equipment) can be monitored, analyzed, and warned [13]. Although visualization technology has developed, it is often only managed for individual safety risk factors. The hoisting operation process of prefabricated buildings is a dynamic system that contains multiple factors of the human-machine-component-environment. Therefore, the elements contained in the system cannot be separated and controlled separately. Digital twin provides a solution to complex system problems and has been widely used in workshop scheduling [14], fault detection, and diagnosis [15] in the field of intelligent manufacturing. Digital twin can integrate various technologies. Based on establishing a virtual model, the data collected from the site are fused with the model, and the analysis results are fed back to the construction site in real-time to realize the visual control of safety risks. However, the application of digital twin in the construction industry is not mature enough. The introduction of a digital twin to the safety management of prefabricated building hoisting mainly faces the following problems: based on considering the correlation between the safety risk factors of prefabricated building hoisting, it is very important to integrate the safety risk mechanism model with the data collected in real-time and achieve the purpose of the scientific decision-making of safety control.

This paper presents a framework for the safety risk control of prefabricated building hoisting based on digital twin. To ensure the better integration of the mechanism model and data to express the relationship between safety risk factors, the hierarchical model of hoisting safety risk is defined in the process of establishing the digital twin model. A multi-level hierarchical model is established by using the Decision-making Trial and Evaluation Laboratory (DEMATEL) and Interpretative Structural Modeling Method (ISM) to show the influential relationship between the factors of hoisting safety risk. Bayesian network is introduced in the process of driving the digital twin mechanism model and monitoring data integration analysis. After determining the direct and indirect inducement variables of security risk, the prior probability of each node is obtained by training Bayesian network parameters with the expectation–maximization (EM) algorithm. If the direct inducement variable is in a dangerous state, the occurrence probability is set to 100% and input into the Bayesian network structure as evidence to obtain the posterior probability. By calculating the criticality of each indirect inducement variable to the direct inducement variable in a dangerous state and comparing the criticality, the key factors leading to the occurrence of danger are screened to achieve the purpose of the visual analysis of safety risk control decision. Finally, the feasibility of the proposed hoisting safety risk control framework is further verified by taking the risk of the cable inclination at a certain time in a specific case as an example.

The organization of the paper is as follows. Section 2 presents an in-depth literature review of safety risk management and digital twin. Section 3 introduces the DT-based prefabricated building hoisting safety risk control framework. Section 4 describes the establishment process of hoisting a safety risk control model based on digital twinning, including the physical model, virtual model, and safety risk hierarchy model. Section 5 introduces the data fusion steps of the DT model driven by the Bayesian network. Section 6 uses a case study to validate the proposed method. Section 7 summarizes the major findings of this study and concludes.

## 2. Literature Review

The literature review of this paper includes two parts: construction safety risk management and digital twin. This section focuses on the development of safety risk management methods and related research on the safety risk management of prefabricated building construction, and analyzes the shortcomings of prefabricated building safety management compared with other fields. In addition, the deep application of digital twin in the field of intelligent manufacturing and related research in the field of intelligent construction is introduced to highlight the advantages of digital twin technology.

### 2.1. Construction Safety Risk Management

Many scholars have studied the issue of safety risk management and have formed many mature methods and technologies [16]. Safety risk management includes safety risk identification and assessment, security risk prediction, and security risk control. At present, work breakdown structure-risk breakdown structure (WBS-RBS) [17], analytic hierarchy process (AHP) [18], fault tree analysis (FTA) [19], Fuzzy comprehensive evaluation (FCE) [20], Cloud model [21], D-S evidence theory [16], and other methods have been used in construction safety risk management. The safety risk management scheme integrated by various methods has also been applied. For example, Yang et al. introduced data-driven Bayesian networks into the TOPSIS method. A new ship detention risk assessment method and reasonable risk control strategy are proposed [22]. With the deepening of theoretical research on safety risk management, the research focus has gradually turned to the essence of safety risk. Based on the extraction of the accident chain, the construction of the risk network, and the formulation of safety measures, Chen et al. proposed a data-driven research framework of construction engineering safety promotion strategy based on the risk network [23].

In recent years, BIM, artificial intelligence, and Internet of Things are also applied to construction safety risk management. Choe et al. proposed a four-dimensional building safety planning process, which solved the problem of spatio-temporal safety information integration at specific locations, and provided an active safety planning tool for safety managers [24]. Lin et al. used the Fuzzy set theory and machine learning method in the risk assessment of mining process and integrated real-time collected data into the BIM management platform to monitor, control, and manage dynamic safety risks [25]. Li et al. proposed the safety risk identification system (SRIS) and early warning system (SREWS) for subway construction in China. The contents of the risk identification knowledge base, retrieval matching algorithm, data fusion, and BIM technology monitoring safety state are mainly studied [26]. Some scholars also apply ontology knowledge [27], computer vision, and semantic reasoning [28] to construction safety management, to more accurately assess the level of safety risks.

In the methods mentioned above, the objects of construction safety risk assessment and control are mostly subway, tunnel, pipe gallery, and other underground projects. Although BIM, Internet of Things, artificial intelligence, and other technologies are integrated, the technologies are not very well integrated, the information interaction mechanism is still imperfect, and most studies ignore the correlation between safety risks. At present, the research on the safety risk management of prefabricated building hoisting operation is relatively minor. Safety risk assessment methods are mostly static and subjective. Xun et al. proposed the DEMATEL method to calculate the mutual influence between the index weights and the method of applying game theory to calculate the combined weights. The safety risk of the prefabricated building hoisting operation is evaluated by the cloud model [29]. Zhao et al., based on the method of determining the weight of the conventional safety risk index, introduced the system dynamics theory and established the SD model of the safety risk measurement of prefabricated building hoisting operation combined with qualitative and quantitative, which solved the dynamic and complex problems in the construction safety risk system [30]. Shen et al. constructed a safety risk assessment model based on Cloud-BN to dynamically evaluate the safety risk of prefabricated hoisting [31]. Liu et al. established a safety risk early warning model of hoisting operation based on RVM (relevance vector machine) to accurately judge the safety status of hoisting operation [32]. In addition, some scholars analyzed the safety accidents of tower cranes and determined the safety risk factors of tower cranes. From the perspective of complex social and technical systems, the safety of tower cranes was evaluated by qualitative and quantitative methods [4], the safety factor of tower cranes was developed [33], and the safety standards were improved [34].

### 2.2. Digital Twin

Digital twinning refers to mapping physical entities in the real world into the digital world, and forming a digital model corresponding to physical entities. Digital models and physical entities can be bidirectional information exchange fusion and iterative optimization, which can improve the performance of physical systems in the real world. The concept of digital twin was first proposed by Professor Grieves in the product life cycle management course of the University of Michigan in 2003 [35]. It was mainly used in the military and aerospace fields in the early period [36,37]. Digital twin is now widely used in intelligent manufacturing. Tao et al. proposed the concept of a digital twin five-dimensional model and applied it to 10 fields, such as satellite/space communication network, ship, vehicle, manufacturing workshop, and smart city [38]. He also proposed the construction theory of the digital twin model [39] and the evaluation index system of the digital twin model [40]. Digital twin technology is suitable for solving complex system problems and has entered a more in-depth research stage of dynamic modeling [41], data fusion [42], and virtual-real interaction [43] in the manufacturing industry.

Scholars in the construction industry have gradually realized that digital twin technology is the key technology to realize intelligent construction [44], which can meet the needs of data fusion and interaction. The application and components of digital twin in the construction industry have not been clearly defined, but some scholars have made preliminary explorations in design, construction, operation, and maintenance in the construction field [45]. Akula et al. realized the real-time monitoring of drilling process hazards by processing and integrating point clouds from 3D imaging technology to the drilling process [46]. Jiang et al. proposed an intelligent safety management framework for construction sites based on the concepts of digital twinning and the CPS system, which can alarm and control dangerous states such as personnel and machinery [47]. Liu et al. proposed an intelligent fire evacuation method based on digital twin, which was verified in the Winter Olympics venues, and laid a foundation for the development of intelligent operation and maintenance [48]. However, these research results have not been confirmed under more complex conditions. In the field of hoisting safety risk management, Liu et al. also proposed a set of modeling theories for the space-time evolution of the prefabricated building construction process, and focused on the modeling method and process [49]. A framework of hoisting safety risk coupling rule mining based on digital twin is also proposed [50]. In summary, the realization of digital twin in the construction field depends on the integrated application of BIM technology and Internet of Things technology [51]. The application of digital twin in the construction stage is in the real-time simulation stage [1]. In the existing research on hoisting safety risk management, the modeling method of digital twin has not risen to the research on the essence of safety risk, and the fusion and interaction mechanism between real-time data and the model has become a key issue of research.

### 2.3. Research Gap

Although many scholars have studied the construction safety management methods in the above literature, there are still some limitations. The safety risk management method integrating BIM, Internet of Things, and other technologies has been applied to the tunnel, subway, and other underground engineering construction fields, and the concept of digital twin has also been introduced. However, the interactive feedback mechanism of safety risk information is still not perfect. The method of real-time warning of safety risk state based on threshold lacks the research on the interaction of safety risk factors and the effective control and decision-making basis. Compared with the safety management methods such as tunnels and subways, the safety management method of prefabricated building hoisting has the limitations of a static state, relying on expert experience, and low intelligence level. The existing modeling methods of digital twin hoisting safety risk management have not risen to the research level of the essence of hoisting safety risk, and there is still a large gap in the research when it comes to how to integrate hoisting safety risk correlation into the establishment of a digital twin model. The introduction of digital twin can integrate BIM, Internet of Things, and other technologies, and establish a virtual–real interaction mechanism through real-time data collection, transmission, and analysis. In the study of the safety management and control of prefabricated building hoisting, how to better integrate the correlation between safety risk indicators and the virtual model is also a problem to be solved. This paper presents a framework for the safety risk control of prefabricated building hoisting based on digital twin. To ensure the better integration of the mechanism model expressing the relationship between the safety risk index factors and the monitoring data, the safety risk hierarchical model is defined in the process of establishing the digital twin model. The Bayesian network is introduced to effectively combine the real-time collected data with the visual network structure, which establishes an effective virtual–real interaction mechanism for the scientific decision-making of the safety risk control of prefabricated building hoisting based on digital twin.

## 3. Digital Twin Framework for Safety Risk Control of Prefabricated Building Hoisting

**Figure 1:**
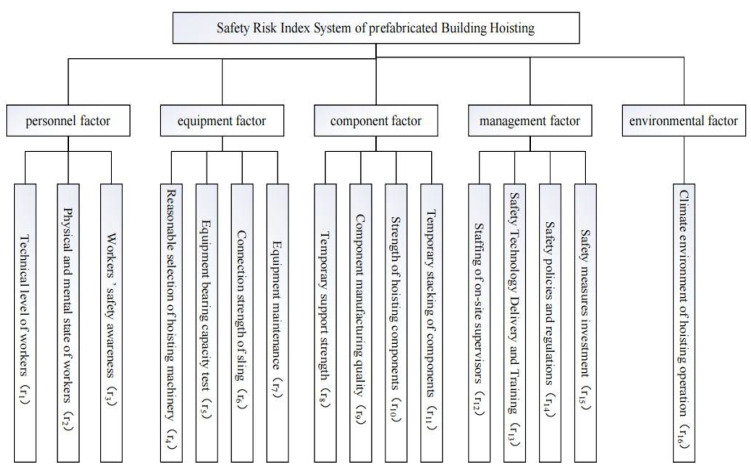
Safety risk index of prefabricated building hoisting.

The establishment of the safety risk index of prefabricated building hoisting is the foundation of the building safety risk control digital twin framework. The safety risk factors of a prefabricated building hoisting operation site include personnel factors, equipment factors, component factors, environmental factors, and management factors. By consulting a large number of research studies [4,29,30,31,32,50] and discussing them with experts, the safety risk index system of prefabricated building hoisting is established, as shown in Figure 1.

**Figure 2:**
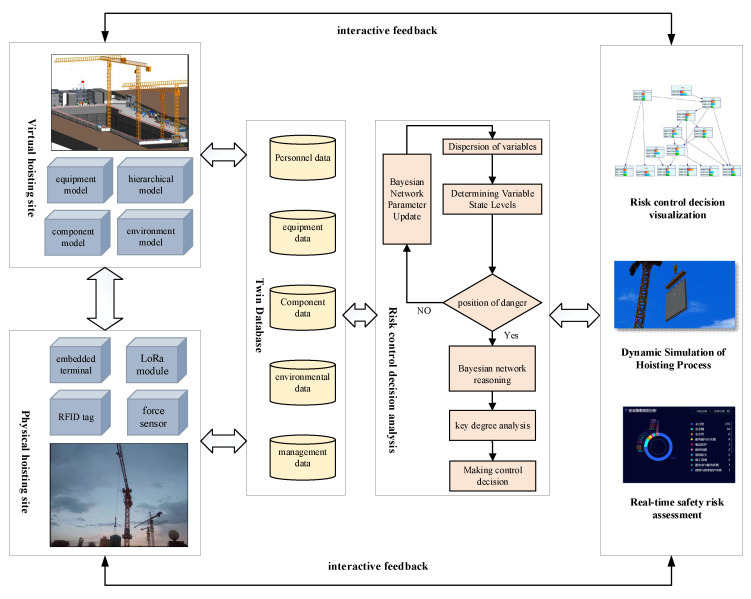
Digital twin architecture for hoisting safety risk control.

The prefabricated building hoisting site is a dynamic and complex system that includes multi-dimensional human-machine-component-environment. When building a digital twin framework for hoisting safety risk control, the idea of dynamic modeling should be integrated based on the geometry-physics-behavior-rule model. Not only the establishment method of each component of the digital twin model, but also the real-time interaction between the model and the data should be considered. In this way, the analysis results of the virtual hoisting site can be fed back to the physical hoisting site in real-time to achieve scientific decision-making. The digital twin framework for the safety risk control of prefabricated building hoisting is shown in Figure 2.

Based on the idea of establishing the digital twin evolution model [52], the hoisting safety risk control framework is built according to the realization logic of the digital twin model. Firstly, Revit software is used to establish the geometric model of the hoisting operation site (equipment, environment, prefabricated components, etc.) and the position relationship between each element on the site is clarified. The assembly and integration of each element model are carried out to complete the high-precision simulation of each element and its attributes, from the physical hoisting site to the virtual hoisting site. Secondly, the influential relationship between safety risk factors in the hoisting process is analyzed, and the hierarchical relationship between risk factors is given to establish a hierarchical model of safety risk. By introducing a Bayesian network, the variable information on safety risk elements collected on-site is discretized and its state-level is judged. The variable information is shown in Table 1. If it is in a dangerous state, the posterior probability is obtained by Bayesian network reasoning, and the key degree of indirect inducing factors to dangerous state variables is calculated to lay the foundation for reasonable decision-making. Finally, to ensure the real-time interaction between the above data and the mechanism model, data acquisition and transmission devices such as RFID tags and LoRa modules are set at the hoisting site to monitor the status of each factor at the hoisting site and upload the data to the twin database for analysis. The established BIM model is lightweight, and processed and loaded on the web page by WebGL technology to display the real-time sensing data and visualization results of decision analysis, and realize the visualization of risk control decision, the dynamic simulation of the hoisting process, and the real-time assessment of safety risk on the physical hoisting site.

## 4. Establishment of Digital Twin Model for Safety Risk Control of Prefabricated Building Hoisting

The digital twin risk model is built from three aspects: the physical space resource allocation of hoisting operation, the virtual space modeling and simulation of hoisting operation, and the hierarchical model of hoisting safety risk, aiming at effective virtual-real interaction and the full integration of the data and model of the digital twin model.

### 4.1. Physical Space Resource Allocation in Hoisting Operation

According to the digital twin framework of hoisting safety risk control, the physical entities in hoisting operation space include workers, tower crane equipment, prefabricated components, and hoisting operation site layout. To better drive the digital twin framework of hoisting safety risk control, it is necessary to connect various elements of human-machine-component-environment in real-time, so that the physical space of the hoisting operation has the virtual and real interaction ability of intelligent perception and transmission. At present, the Internet of Things technology system composed of a multi-sensor, radiofrequency technology and a wireless network has been widely used, which provides support for the virtual-real interaction of the digital twin model. The data variables that can be monitored in the physical space of hoisting operation according to the safety risk index system are shown in Table 1.

According to the physical space modeling method [50] proposed by the research group, for example, active RFID tags can be used to locate the position of personnel and components, and the relevant basic information of personnel and components is input into the tags for subsequent data retrieval. The wind speed, the stress and strain of components and tower cranes, and the inclination change of hoisting components can be collected through embedded sensors. Different information perception schemes are selected according to the variable information required for monitoring in actual projects.

The collected monitoring data can be connected to the hoisting operation space resources through a communication protocol. Combined with the configured intelligent sensing device and wireless transmission network, the full interconnection and integration of multi-source heterogeneous elements such as human, machine, component, and environment in the physical workshop can be realized.

### 4.2. Virtual Space Modeling and Simulation of Hoisting Operation

**Figure 3:**
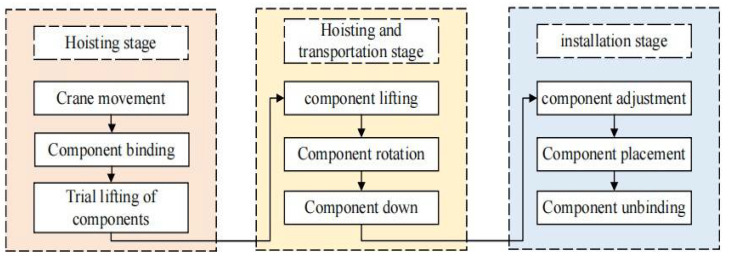
Division of prefabricated building hoisting process.

The hoisting process mainly includes the hoisting stage, hoisting transportation stage, and installation stage of the component. The specific process is shown in Figure 3. The hoisting process is a complex, continuous, and dynamic process. To ensure the high fidelity of the digital twin model, this paper incorporates the modeling idea of “geometry-logic” into the existing virtual model establishment method of the research group [54]. According to the characteristics of various entities in the physical space, modeling is carried out from two dimensions of geometry and logic. The geometric model mainly includes the personnel attributes of space, tower crane equipment, prefabricated components, and field working environment layout in the physical space. Specifically, the geometric model established by BIM technology should reflect the appearance, size information, attribute information of components, equipment, hoisting site layout, and the assembly relationship between various entity elements. The logical model mainly drives the consistency between the virtual space of hoisting operation and the physical space in behavior, state, and operation in the form of data, such as the working state of hoisting equipment and the state of prefabricated components. To realize the faithful mapping of virtual space to physical space, BIM4D technology can be used to add time factors to define the logical model containing factors such as behavior, rules, and constraints; for example, the position change of the component. The geometric model and logical model are combined to form a completely virtual space model of hoisting operation.

The BIM model can provide a visualization platform for the virtual space of the hoisting operation. The BIM model is lightweight, and processed and mounted on the web page, and embedded in the data analysis and processing module with intelligent algorithm as the core. On this basis, the virtual and real information interaction relies on the active RFID tags, sensors, and wireless transmission networks configured in the hoisting site to collect real-time data for the dynamic change process of human-machine-component-environment and integrate them. After the conversion of the gateway communication protocol, the data are sent to the virtual space by a wireless network. The virtual space based on the lightweight BIM model visually displays, analyzes, and feeds back the decision results to realize the real-time and effective control of the safety risk of the physical space of the hoisting operation.

### 4.3. Construction of Hoisting Safety Risk Hierarchical Model

There are many factors influencing hoisting safety risks. There is a certain correlation between the various factors, and the strength of the correlation effect is also different. In this paper, a hierarchical model of hoisting safety risk is added to the original digital twin model, to clearly express the direct and indirect causes of safety accidents, ensure the better integration of the data and virtual model, and support better scientific control decisions. The integration of the DEMATEL method and the ISM method to determine the hierarchical structure of safety risk can effectively determine the causal relationship between factors and obtain the deep-seated factors leading to accidents. This lays the foundation for subsequent expert knowledge and real-time data fusion. The idea for the DEMATEL-ISM method is to obtain the comprehensive influence matrix based on the DEMATEL method. In view of the influence of factors on the ISM method, it is used to obtain the overall influence matrix, and the reachable matrix is obtained by optimizing the given threshold. Finally, the hierarchical structure between accidents is divided, and the relationship between accident systems is obtained. The specific steps are as follows:

1. According to the safety risk index system of prefabricated building hoisting proposed above, the influence levels of safety risk factors are divided. The index system set is denoted as *A*. Questionnaires were distributed to three experts, namely technical research experts of prefabricated construction, enterprise managers, and project managers in the field. The influence relationship between the two indicators is denoted as *β*. The questionnaire results from the three experts are further discussed to comprehensively determine the direct impact matrix *B* between risk factors.
2. The initial direct influence matrix *B* is normalized according to Equation (1) to obtain the normalized matrix *C*.

(1) $$C=\frac{1}{\underset{1\leq i\leq n}{\max}\sum_{j=1}^{n}\beta_{ij}}B$$

In the formula, $\underset{1\ll i\ll n}{\max}\sum_{j=1}^{n}\beta_{ij}$ is the row sum maximum of the direct influence matrix.

3. The normalized matrix is processed according to Equation (2) and the comprehensive influence matrix *T* is obtained.

(2) $$T=C{\left(I,-,C\right)}^{-1}$$

Based on the comprehensive influence matrix, the overall influence matrix *H* is obtained according to Equation (3). *I* is the unit matrix.

(3) H=I+T

4. Referring to the method of λ in Reference [55], the threshold λ is set to 0.1 and the reachable matrix *K* = (*k_ij_*)nxn is obtained. A reachable matrix indicates whether there is a connection path from one element to another. *k_ij_* is the element in the reachability matrix and *h_ij_* is the element in the comprehensive influence matrix.

(4) $$k_{ij}=\left\{\begin{array}{cc} 1 & h_{ij}\geq \lambda \\ 0 & h_{ij}<\lambda \end{array}\right.i,j=1,2,\cdot \cdot \cdot ,n$$

*k_ij_* = 1 indicates that factor *i* has a direct impact on factor *j*, and *k_ij_* = 0 indicates that there is no direct impact between the two factors.

5. Based on the reachable matrix, the reachable set and the antecedent set of factors are constructed, as shown in Equation (5).

(5) $$\begin{array}{l} R_{i}=\left\{r_{j}|r_{j}\in R,k_{ij}\neq 0\right\}i=1,2,\cdot \cdot \cdot n\\ S_{i}=\left\{r_{j}|r_{j}\in R,k_{ji}\neq 0\right\}i=1,2,\cdot \cdot \cdot n \end{array}$$

In the formula, *R_i_* is the reachable set of each factor. *S_i_* is the antecedent set of each factor. *R* is the correlation matrix of the prefabricated building hoisting safety risk index. *r_j_* is the hoisting safety risk index.

**Figure 4:**
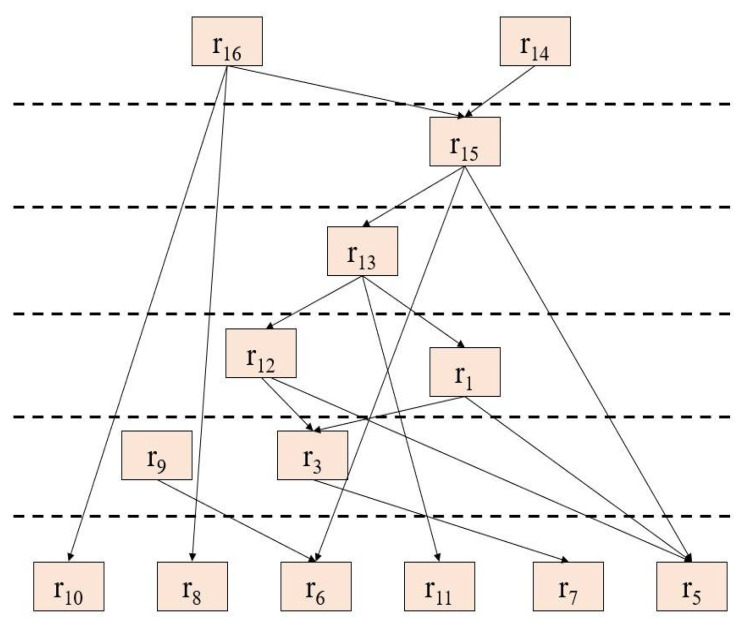
Hierarchical model of hoisting safety risk factors.

6. Equation (6) is used to verify each factor. If it is established, it is determined as the first layer factor, and the corresponding rows and columns are divided in the reachable matrix. The above steps are repeated to determine the level of other factors in turn, until all the factors in the reachable matrix are divided. Thus, the safety risk hierarchy model is determined, as shown in Figure 4
  (6) $$R_{i}=R_{i}\cap S_{i},i=1,2,\cdot \cdot \cdot ,n$$.

## 5. Safety Risk Control Method of Digital Twin Hoisting Driven by Bayesian Network

Based on the above digital twin model of hoisting safety risk control, the configuration of intelligent resources in the physical space of hoisting operation can ensure the real-time acquisition and transmission of data. The establishment of a virtual space model lays a foundation for the visualization of risk control decision-making and the dynamic monitoring of the hoisting process. However, integrating data and virtual models requires artificial intelligence algorithms to drive it. Considering the correlation between safety risks and incomplete safety monitoring mechanisms, this paper introduces a Bayesian network to drive a better combination of digital twin model and data.

### 5.1. Overall Operation Mechanism of Hoisting Safety Risk Control Method

**Figure 5:**
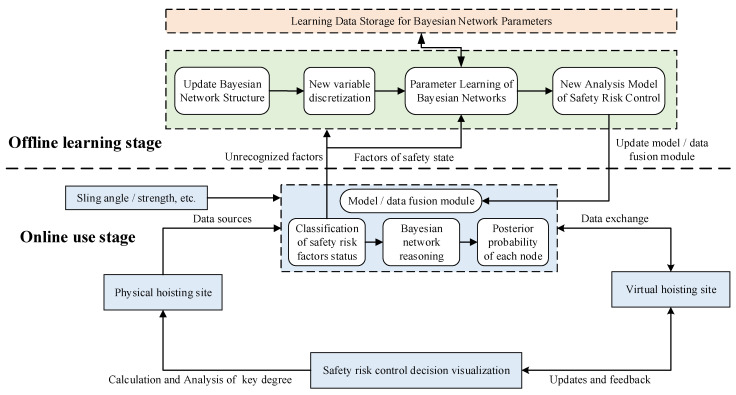
Overall operation mechanism of safety risk control method for hoisting.

Based on the establishment of the digital twin model, this paper fuses the digital twin model and data through the Bayesian network, as shown in Figure 5. The safety risk management of prefabricated building hoisting includes multiple dimensions of human-machine-component-environment. It has great advantages to solve the complex system of the safety risk management of prefabricated building hoisting by using the real-time and dynamic characteristics of digital twin technology. Considering the relationship between safety risks and the absence of a large number of historical data on safety risk management, the graph model is undoubtedly an effective way to solve the problem. The graph model is one of the most popular methods in the field of machine learning. It is composed of directed or undirected graphs through a series of nodes and wires. In the field of solving construction safety problems, the Bayesian network, as a kind of graph model algorithm, has been widely used. The establishment of its network structure can show a correlation between safety risk factors. Bayesian network reasoning can be used to assist decision-making, providing an internal driving force for hoisting safety risk control method based on digital twin.

The overall operation mechanism of the hoisting safety risk control method is mainly divided into the offline learning stage and the online use stage. In the online use stage, digital twin technology is used to establish the physical hoisting site and the virtual hoisting site, and the model/data fusion module is configured. The bridge between the physical hoisting site and the virtual hoisting site is established, and the data of the virtual hoisting site are interconnected. The variable information, such as sling angle and strength collected from the physical site, is uploaded to the virtual hoisting site through intelligent perception and transmission equipment. At the same time, the Bayesian network is used to process the perception data in the model/data fusion module, including the state classification of safety risk factors and the Bayesian network reasoning to calculate the posterior probability of each node. When the Bayesian network model cannot determine and analyze the perceived data, it is judged as a new hoisting safety risk factor index, and the data need to be reprocessed in the offline learning stage. The main work of the offline learning stage is to establish the hoisting safety risk control model based on the Bayesian network. The specific process includes the determination of the Bayesian network structure, the determination of nodes, the discretization of variables, and the learning of Bayesian network parameters. The safety risk factors that cannot be identified and processed in the online use stage are combined with the original Bayesian network structure model to form a new Bayesian network hoisting safety risk control model and update the model and data fusion module. In this process, it is necessary to store the data of the Bayesian network parameter learning for subsequent calls. Through data analysis and calculation, the key degree is obtained. The safety risk control strategy is formed and fed back to the physical hoisting site. The fusion of model and data is realized, and the safety of the hoisting site is effectively guaranteed.

### 5.2. Specific Process of Hoisting Safety Risk Control Analysis

The hoisting operation is a complex, discrete, and irregular system, so it is difficult to establish the corresponding mathematical model. The amount of data on the construction site is larger, and the construction monitoring data are difficult to retain and form a historical database with. Based on the above characteristics of the hoisting operation site, the Bayesian network (BN) is selected to drive the digital twin model. BN has been used in fault diagnosis, abnormal condition identification, and other research fields. It has good advantages in the expression of nonlinear and uncertain relations.

**Figure 6:**
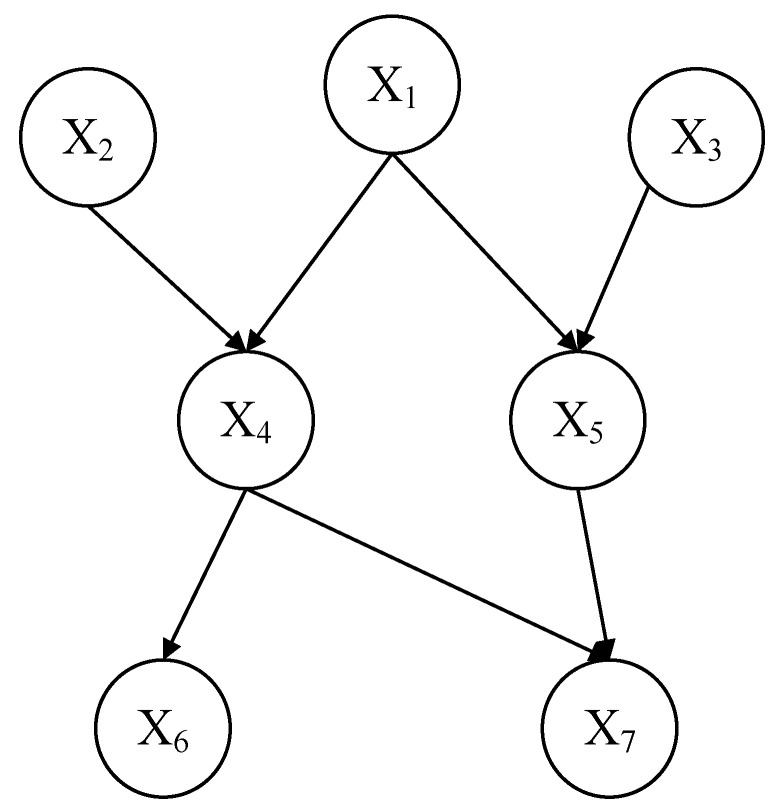
Bayesian network topology.

BN is an uncertain causal correlation model with strong reasoning ability. The definition of a Bayesian network can be expressed in the form of a tuple: G=(V,E) means directed acyclic graphs. V represents the set of nodes in a directed acyclic graph. E represents the directed edge connecting two related nodes in the directed acyclic graph, indicating qualitative information. θ refers to the probability distribution between variables, which is essentially a conditional probability table, representing quantitative information. The Bayesian network topology is shown in Figure 6. Taking node X_4_ as an example, X_1_ and X_2_ are the parent nodes of X_4_, and X_4_ is affected by X_1_ and X_2_. Under the premise of given evidence, the Bayesian network can update the probability of variables by probability propagation or reasoning under limited, incomplete, and uncertain conditions.

BN is quantitatively analyzed by the D-separation criterion and chain rule. All root nodes are conditional independent according to the D-separation criterion, and other nodes depend only on their direct parent nodes. According to conditional independence and chain rule, BN denotes the joint probability distribution *P*(*U*) of variables *U* = {*A*_1_, *A*_2_…, *A_n_*} contained in the network (as shown in Equation (7)).

(7) $$P(U)=\prod_{i=1}^{n}P(A_{i}|Pa(A_{i}))$$

*Pa* (*A_i_*) is the parent node of *A_i_* in BN. When new information is given, called evidence E, BN can update the prior probability of the event by reasoning engine (as shown in Equation (8)).

(8) $$P(U|E)=P(U,E)/P(E)=P(U,E)/\sum_{U}P(U,E)$$

Aimed at the specific scene of prefabricated building hoisting safety risk control, the operation process of the digital twin safety risk control framework driven by the Bayesian network is as follows:

(1) Step 1: Transform the hierarchical model determined by the DEMATEL-ISM method in Section 2.3 into a Bayesian network structure, which requires transforming the quantifiable index r_i_ in the original hierarchical model into the corresponding variables. The bottom layer of the hierarchical model is divided into direct incentive variables, and the other layers are divided into indirect incentive variables.
(2) Step 2: According to the Bayesian network structure, the network nodes are graded by expert scoring and the continuous data are discretized, as shown in Table 2 and Table 3.
(3) Step 3: After the Bayesian network structure is determined, it is necessary to determine the Bayesian network parameters. The goal of Bayesian network parameter learning is to determine the conditional probability of nodes in the Bayesian network model by using prior knowledge combined with the given network topology and training sample set. The expectation–maximization algorithm (EM algorithm) is applied to Bayesian network parameter learning based on the above data processing characteristics.

Existing security information monitoring mechanisms are incomplete, resulting in no large database or complete historical data to refer to. Relying on expert experience is too subjective. The EM algorithm is a general algorithm for calculating maximum likelihood function (ML), which is mainly used for Bayesian network parameter learning under incomplete data. The obtained data samples of hoisting safety risk indicators are recorded as $D=\left\{D_{1},D_{2},\cdot \cdot \cdot ,D_{m}\right\}$. Each sample $D_{i}$ is composed of unknown variables $X_{i}$ and observed variables $Y_{i}$. The probability distribution function $Q(X=x_{i}|Y)$ of the residual variable X is defined, which represents the probability of $X=x_{i}$ when the observation value is $Y_{i}$. It means $\sum Q(X=x_{i}|Y)=1$.

Any sample $D_{i}$ is composed of unknown variables $X_{i}$ and observation variables $Y_{i}$. At this time, it is assumed that the likelihood function L is shown in Equation (9).

(9) $$L(\theta )=\sum_{i=1}^{m}\log\sum P(X=x_{i},Y=D)$$

Assuming that Q(X|Y) is a convex function with extreme values, according to Jensen inequality:

(10) $$\begin{array}{l} L\left(\theta \right)=\sum_{i=1}^{m}\log\sum_{n}Q(X|Y)\times \frac{P(X=x_{i},Y=D)}{Q(X|Y)}\\ \geq \sum_{i=1}^{m}\sum_{n}Q(X|Y)\times \log\frac{P(X=x_{i},Y=D)}{Q(X|Y)} \end{array}$$

The EM algorithm mainly calculates the expected value of the log-likelihood function according to Q(X|Y) and continuously optimizes the Q(X|Y) value to find the L value that satisfies the maximum value of the log-likelihood function. The EM algorithm is a continuous iteration process described above [56].

(4) Step 4: According to the data sample, the EM algorithm is used to solve the prior probability and conditional probability of each node.
(5) Step 5: If the direct inducement variable is in a dangerous state, the probability of the occurrence of the state at this time is changed to 100%, and as evidence, information is input into the Bayesian network model for reasoning, and continued in Step 6. If there is no danger, the data are stored in the offline learning stage to update the prior probability.
(6) Step 6: Based on the results of Bayesian network reasoning, the key degree of indirect incentive variables to direct incentive variables is calculated, which makes the factors that need to be controlled clearer, as shown in Equation (11).

(11) $$X_{t}(C_{k})=\frac{1}{p_{k}}\sqrt{\sum_{c_{k}}^{p_{k}}{\left(P^{\prime }(C_{k}=c_{k}|R=r)-P(C_{k}=c_{k})\right)}^{2}}$$

$p_{k}$ represents the number of discrete states of risk indicators. $P^{\prime }(C_{k}=c_{k}|R=r)$ represents the posterior probability of $C_{k}$ in $c_{k}$ state when node *R* is in *r* state; $P(C_{k}=c_{k})$ denotes the prior probability of $C_{k}$ in $c_{k}$ state.

(7) Step 7: In the process of data input into the Bayesian network structure, it is necessary to determine whether new anomalies occur. If they do occur, Step 1 is entered to re-determine the Bayesian network structure and update the Bayesian network model offline.

## 6. Case Study

Taking a prefabricated building project in Tianjin as an example, this paper verifies the methods mentioned in the article. The prefabricated building project is located in the leisure area of Huanggang, Tianjin Binhai New Area. The total building area is 71,172.43 square meters. The structural type is a reinforced concrete shear wall structure and some prefabricated structural members are used. The project has an assembly rate of 37.5%, a maximum height of 54 m, and a maximum number of 18 floors. The prefabricated parts of the prefabricated project are prefabricated composite floor slabs, prefabricated stair slabs, and partially prefabricated shear walls.

**Figure 7:**
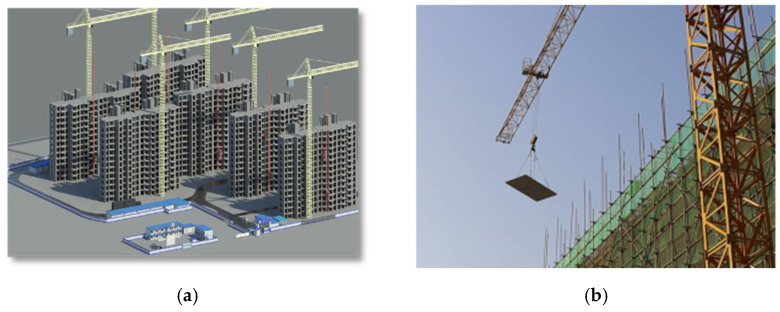
Construction site of the project. (**a**) BIM model of construction project; (**b**) On-site construction.

This project has problems such as prefabricated component management and safety risk aversion. The construction site involves many tower cranes and many types of components. The relationship among equipment, components, and environment is complex. The construction site is shown in Figure 7. Therefore, it is necessary to study the safety risk management in the hoisting process of this project to prevent and control the safety risk.

### 6.1. Establishment of Digital Twin Model

On the level of physical entity modeling, intelligent sensing and data transmission equipment are mainly configured on the four dimensions of the human, machine, component, and environment of the hoisting site. Active RFID tags are embedded in the prefabricated components to store the ID and basic attributes of the components. RFID tags are also configured on the worker’s safety hat to store the identity and location information of the personnel. To read the inclination, strength, position, and other information of the component, IMU (inertial measurement unit) and mechanical sensors are installed on the component. However, the information needed for tower crane equipment monitoring (load-weight ratio, wind speed, height, amplitude, etc.) is more and more complex. Therefore, mechanical sensors, wind speed sensors, height sensors, and other types of sensors are mainly used for data acquisition. RFID readers are also configured to obtain the position and attribute information of personnel and components in real-time. The sensor accuracy or error used above is shown in Table 4. In the process of data acquisition, considering the process of hoisting activities and the real-time nature of digital twin, the time interval of sensor data collection is uniformly set to 90 sec, and the redundant data are deleted, which is also convenient for data analysis at the same time, and more accurate results are obtained.

**Figure 8:**
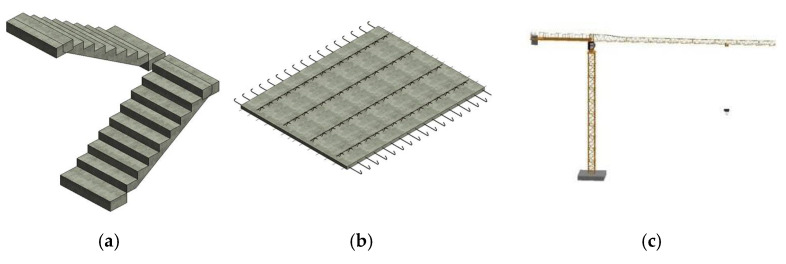
Prefabricated components and tower crane equipment model. (**a**) BIM model of prefabricated stairs, (**b**) BIM model of composite board, (**c**) BIM model of tower crane.

On the level of virtual hoisting site modeling, considering the dynamic hoisting process, the virtual model with high fidelity is built by BIM technology. The overall model of the hoisting site is divided into the equipment model, prefabricated component model, personnel model, and hoisting environment model, and each model is further divided in detail. Taking the establishment of the hoisting environment model as an example, it is necessary to consider the display of site layout and the stacking of prefabricated components. Based on establishing their geometric models, Revit software is used to couple with the environmental model to form a digital twin model, with high fidelity according to the position relationship, assembly relationship, and construction process relationship between equipment, prefabricated components, and the environmental model. At the same time, the models such as equipment and prefabricated components are parameterized, and a family library is established to realize the call of subsequent modeling of the same scene, and realize rapid modeling, as shown in Figure 8. The modeling of the equipment model and component model is the same as above. 

Considering the specific scene of safety risk control in the hoisting process, to better integrate data with the digital twin model, a hierarchical model of safety risk is added to characterize the correlation between various hoisting safety risks. The security risk hierarchy model established in Section 2.3 is generally applicable and can be directly used in this case.

**Figure 9:**
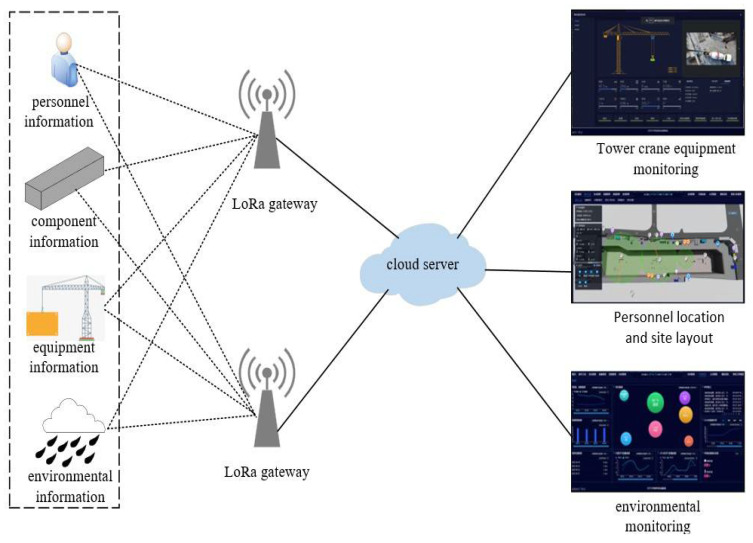
LoRa network architecture.

On the level of virtual-real interaction correlation modeling, the important task is to establish the bridge of information interaction between the physical hoisting site and the virtual hoisting site. Virtual-real interaction modeling mainly includes two aspects: one is data collection and transmission; another is the packaging service for digital twin models. In this case, LoRa technology with low-power consumption, long-distance and low cost is used to solve the problem of connecting a large number of sensors to the gateway, and a star topology is built. The data collected from the physical hoisting site are uploaded to the LoRa gateway. The LoRa gateway uploads the data to the cloud server through the 4G network, and the local server and customer end can access the site information through the cloud server, as shown in Figure 9.

**Figure 10:**
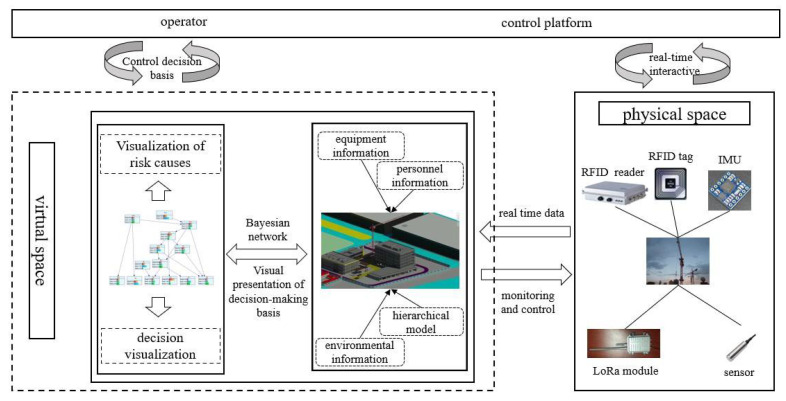
Operating mechanism of digital twin holistic model.

The operation mechanism of the overall digital twin model of this project and the partial virtual hoisting site model are shown in Figure 10. Taking the whole BIM model as a data carrier and visualization platform, the collected data are uploaded through the data acquisition and transmission system of physical space. After judging the safety risk state, the node probability of each variable in the Bayesian network is updated. The key cause analysis of the dangerous state nodes and the feedback of the results are displayed on the BIM platform to realize the visual analysis and decision-making of safety risks, avoid the blind judgment of the causes of safety risks, and thus effectively control the safety risks.

### 6.2. Safety Risk Control Method of Digital Twin Hoisting Driven by Bayesian Network

This paper proposes a safety risk control method for prefabricated building hoisting based on digital twin, focusing on the fusion mechanism of the digital twin model and field monitoring data. Therefore, this part focuses on the introduction of the Bayesian network to realize the visualization of the risk decision analysis method process and verify the feasibility of the method.

#### 6.2.1. Data Preparation

Based on the digital twin model of prefabricated building hoisting safety risk control, according to the method of data perception and transmission on the physical hoisting field level, the data collection of each safety risk index variable is carried out at the construction site. Due to the high repeatability of the data collected in one day, we further integrate the data collected for many consecutive days to obtain the data at three different times, as shown in Table 4. To ensure the accuracy of Bayesian network parameter learning, according to the opinions of experts, the integrated data samples are further copied and expanded. For the three-state variables in the Table 5, 0,1,2 respectively represent the safety state of the index in good, general, and poor. For 2 state variables, 0 and 1 represent better and worse, respectively.

#### 6.2.2. Visualization Analysis of Security Risk Control Decision-Making

**Figure 11:**
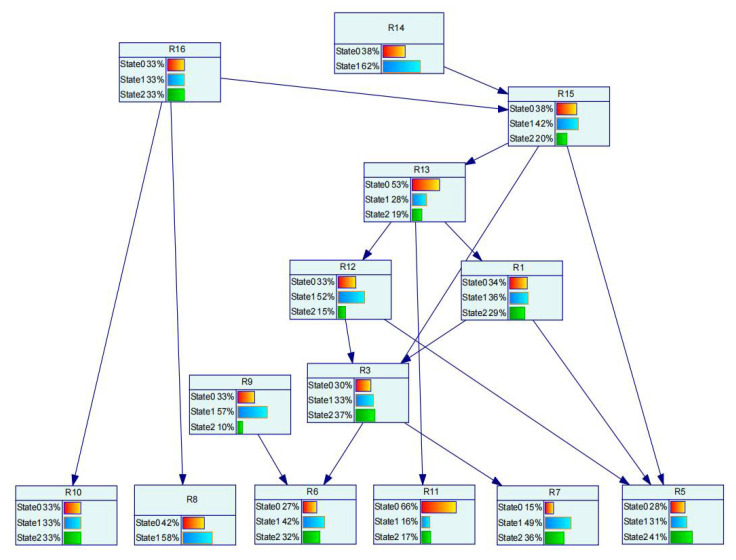
Prior distribution of nodes *.

On the visualization analysis of safety risk control decisions, this paper uses GeNIe software to visualize the Bayesian network. Combined with the safety risk level model described in Section 2.3, the Bayesian network structure is built. The ID of each node in the network structure and the number of their states are defined. The EM algorithm is embedded in GeNIe software. The data collected in Section 6.2.1 are input to learn the Bayesian network node parameters to obtain the prior probability of each node, as shown in Figure 11.

**Figure 12:**
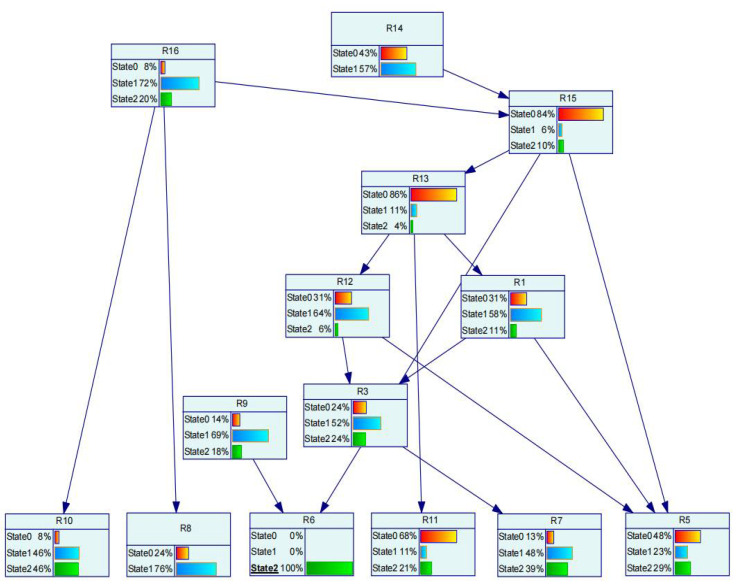
The posterior probability distribution of each node when the sling angle is in a dangerous state *.

At time 3 in Table 5, it can be seen that factor r_6_ is in a dangerous state. The probability of r_6_ in a dangerous state is set to 100% and input into the Bayesian network structure as evidence. The posterior probability of other nodes is obtained when the sling angle is in a dangerous state during hoisting, as shown in Figure 12. At this time, the principle of maximum membership is used to determine the state level of each node. When r_6_ is in a dangerous state, the state of the associated indirect inducing factors is consistent with the state of the data that we collected. Equation (11) is used to solve the key degree $X_{t}(C_{k})$ of the indirect inducing factors associated with r_6_ to the risk factor r_6_, as shown in Table 6. By comparing the values of criticality, the path of safety risk causes is analyzed, and the safety risk control decision is given with emphasis and rationality.

* Note: The uppercase letters representing the node number in Figure 11 and Figure 12 correspond to the lowercase letters in Table 5 and Table 6, and the uppercase letters in the figure are used to distinguish them from numbers.

According to the key degree calculated in the above table, the order of the key degree of indirect inducing factors associated with r_6_ is as follows: Safety measures cost investment ratio r_15_ > wind velocity r_16_ > ratio of participants in safety clearance r_13_ > workers’ safety awareness r_3_ > technical level of workers r_1_ > quality of component production r_9_ > on-site supervision staffing level r_12_ > safety policies and regulations r_14_. The input of safety risk measures and the disturbance of the environment have a great influence on the strength of the connection parts of the hoisting components. In the hoisting site, attention should be paid to whether the wind speed is suitable for the hoisting operation, and whether the input of safety measures meets the requirements. When formulating safety strategies, attention should be paid to emphasizing safety awareness to workers and strengthening the control of the number of participants in the disclosure of safety technology to improve the technical level of workers. At this time, the obtained data will be stored in the offline learning stage of the Bayesian network and used for the next risk state identification and decision analysis.

After the above analysis and processing of the data related to the safety risk factors of hoisting operation, the analysis and processing results will be displayed on the BIM-based visualization platform. Taking the abovementioned factor r_6_ as an example, the platform interface will show that r_6_ (cable inclination) is in a dangerous state, and the position is marked in the BIM model. The risk-causing paths are listed, and the key causes leading to the occurrence of factor r_6_ are analyzed by calculating the key degree. After receiving the risk factors information, the hoisting site management personnel formulate the safety risk control scheme and feed it back to the site operation personnel to rectify the site. The analysis data related to the risk factors will be stored in the cloud database, and the safety risk control measures formulated by the management personnel will also be uploaded to the platform for recording, to serve as a follow-up call reference and improve the efficiency of safety risk management.

## 7. Conclusions and Future Works

With the development of information technology such as BIM, Internet of Things, and artificial intelligence, the construction safety management mode has changed from passive management to active management and real-time monitoring. In the safety management of underground engineering such as subway and tunnel, the integrated application of BIM and Internet of Things technology has been formed, but there is still a lack of effective information interaction mechanism and it ignores the interaction between safety risk factors. In the field of prefabricated building hoisting safety risk management, most research focuses on the importance evaluation of safety risk indicators, mainly relying on expert experience. In the existing research on hoisting safety risk management based on digital twin, the modeling method of digital twin has not risen to the research on the essence of safety risk. On the basis of considering the correlation between safety risk factors, the interaction mechanism of the real-time data and model needs to be improved.

This paper presents a framework for the safety risk control of prefabricated building hoisting based on digital twin. To ensure the better integration of the digital twin model and data, the DEMATEL-ISM method is used to establish the hierarchical model of hoisting safety risk in the process of establishing the digital twin model, so as to express the correlation and risk transfer relationship between safety risks. The Bayesian network is introduced to visualize the risk control decision model based on digital twin. After determining the direct and indirect incentive variables of safety risks, the prior probability of each node is obtained by training the Bayesian network parameters through the EM algorithm. If the direct inducement variable is in a dangerous state, the occurrence probability is set to 100% and input into the Bayesian network structure as evidence to obtain the posterior probability. The key degree of each indirect incentive variable to the direct incentive variable in a dangerous state is calculated, and the safety risk control decision is made by comparing the key degree. The method has been verified in a large prefabricated residential project, which proves the value and effectiveness of the proposed method. The main contributions of this study are as follows:

(1) A complete framework for the safety risk control of prefabricated building hoisting based on digital twin is established. The hoisting activities of prefabricated buildings are analyzed in detail, and the hoisting safety risk index system is sorted out. Aiming at the safety risk factors of hoisting, a digital twin model is established from the aspects of the physical hoisting process, virtual hoisting process, and virtual-real interaction, and the interaction mechanism of each component of digital twin is expounded.
(2) The research on digital twin in the field of prefabricated building hoisting is raised to the level of the research on the essence of safety risk. This paper fully considers the correlation of hoisting safety risk indicators. The hierarchical model of safety risk is added to the digital twin modeling, and the cause path of hoisting safety risk is intuitively expressed. The fusion mechanism of the safety risk mechanism model and real-time monitoring data is established by introducing the Bayesian network, which lays the foundation for the real-time visualization analysis of hoisting safety risk.
(3) The proposed hoisting safety risk control method based on digital twin is verified in practical engineering projects. Through the BIM visualization platform, the location of risk factors, the attribute information of factors themselves, and the cause information are visualized. It is convenient for hoisting site managers to clearly analyze the safety risk state and effectively formulate safety risk control measures, avoid the occurrence of safety risks, and fill in the blanks of the safety risk control decision-making module in the hoisting safety risk management platform.

This study provides a good solution for a visual analysis of safety risk control decisions of prefabricated building hoisting, and solves the problem that the digital twin model cannot effectively integrate with real-time monitoring data under the condition of considering the correlation between safety risks. There are still some limitations to this method. The existing training sample set is limited, and the intelligent decision system supporting hoisting safety risk control is lacking. In addition, how to integrate this method with prefabricated building hoisting safety risk assessment and prediction methods needs further exploration. In future research, we will continue to expand the scale of data samples to improve the accuracy of the hoisting safety risk control method based on digital twinning, and continuously improve the visual display effect and functional modules of the control platform based on BIM technology.

## Figures and Tables

**Table 1:** Monitorable variables of hoisting space [53].

Safety Risk Indicator (r_i_)	Monitorable Variables
Technical level of workers (r_1_)	basic information of workers
Physical and mental state of workers (r_2_)	blink frequency/facial features of tower crane drivers
Workers’ safety awareness (r_3_)	workers’ position/wearing of protective equipment
Equipment bearing capacity test (r_5_)	stress or strain of load/key parts of tower crane/verticality of tower crane
Connection strength of sling (r_6_)	the inclination angle of the hanger/the stress and strain of the hanger
Temporary support strength (r_8_)	stress and strain of supporting key parts
Component manufacturing quality (r_9_)	component basic information
Strength of hoisting components (r_10_)	stress and strain at lifting point of hoisting components
Temporary stacking of components (r_11_)	component location information (whether secondary handling is required)
Climate environment of hoisting operation (r_16_)	wind grade/wind speed

**Table 2:** 2 Risk state division of state discrete variables.

Index	Corresponding Variables	S_0_	S_1_
r_8_	Stability of temporary support	stable	unstable
r_14_	Completeness of safety standards	complete	incomplete

(S_0_ and S_1_ are used to represent non-risk free and risky).

**Table 3:** 3 Risk state division of state discrete variables.

Index	Corresponding Variables	Risk State Division		
		S_0_ (Good)	S_1_ (General)	S_2_ (Poor)
r_1_	Technical level of workers	[90,100)	[60,90)	[0,60)
r_3_	Workers’ safety awareness	[90,100)	[60,90)	[0,60)
r_5_	Actual load ratio	[0,80)	[80,100)	[100,150)
r_6_	Sling angle/°	[30,40)	[40,50)	[50,60)
r_7_	Wear rate of hoisting equipment (%)	[0,10)	[10,40)	[40,50)
r_9_	Quality of Component Production	[90,100)	[60,90)	[0,60)
r_10_	Strength of hoisting components (MPa)	[0,4)	[4,9)	[9,13.5)
r_11_	Temporary stacking of components	[90,100)	[60,90)	[0,60)
r_12_	On-site supervision staffing level	[90,100)	[60,90)	[0,60)
r_13_	The ratio of participants in safety clearance (%)	[90,100)	[60,90)	[0,60)
r_15_	Safety measures cost investment ratio (%)	[3,5)	[1.5,3)	[0,1.5)
r_16_	wind velocity (m·s^−1^)	[0,7.9)	[7.9,10.8)	[10.8,16)

**Table 4:** Accuracy or error of various sensors.

Sensor Type	Accuracy/Error
RFID tag	detection accuracy: 3–5m
IMU (inertial measurement unit)	accuracy: ±0.1
stress and strain sensor	system uncertainty: ≤0.2% ± 1 µε
Tower crane black box (height sensor, wind speed sensor, rotary sensor, weight sensor, inclination sensor, amplitude sensor)	System comprehensive error: ≤0.5%

**Table 5:** Data samples.

	Time 1	Time 2	Time 3
r_1_	0	2	1
r_3_	0	2	1
r_5_	1	2	2
r_6_	0	1	2
r_7_	1	2	1
r_8_	0	1	1
r_9_	0	1	1
r_10_	0	2	1
r_11_	0	0	0
r_12_	0	1	1
r_13_	0	1	0
r_14_	0	1	1
r_15_	1	1	0
r_16_	0	2	1

**Table 6:** The key degree calculation.

Code	Risk Factor	Prior Probability			Posterior Probability			*X_t_*/10^−3^
		S_0_	S_1_	S_2_	S_0_	S_1_	S_2_	
r_1_	Technical level of workers	0.34	0.36	0.29	0.31	0.58	0.11	95.277
r_3_	Workers’ safety awareness	0.53	0.28	0.19	0.24	0.52	0.24	126.579
r_9_	Quality of Component Production	0.33	0.57	0.10	0.14	0.69	0.18	79.512
r_12_	On-site supervision staffing level	0.33	0.52	0.15	0.31	0.64	0.06	50.442
r_13_	Ratio of participants in safety clearance (%)	0.53	0.28	0.19	0.86	0.11	0.04	133.458
r_14_	Safety policies and regulations	0.38	0.62	-	0.43	0.57	-	35.355
r_15_	Safety measures cost investment ratio (%)	0.38	0.42	0.20	0.84	0.06	0.10	187.380
r_16_	wind velocity (m·s^−^^1^)	0.33	0.33	0.33	0.08	0.72	0.20	160.381

## Data Availability

Data sharing not applicable.
